# Supplementary material for: Radiophysiomics: Brain Tumors Classification by Machine Learning and Physiological MRI Data
Source: Cancers (Basel). 2022 May 10;14(10):2363. doi: 10.3390/cancers14102363 (PMC9139355; doi:10.3390/cancers14102363)
Supplement: Supplementary file 1 [file cancers-14-02363-s001.zip › cancers-1716532-SM.pdf]

# Supplementary Materials: Radiophysiomics: Brain Tumors Classification by Machine Learning and Physiological MRI Data

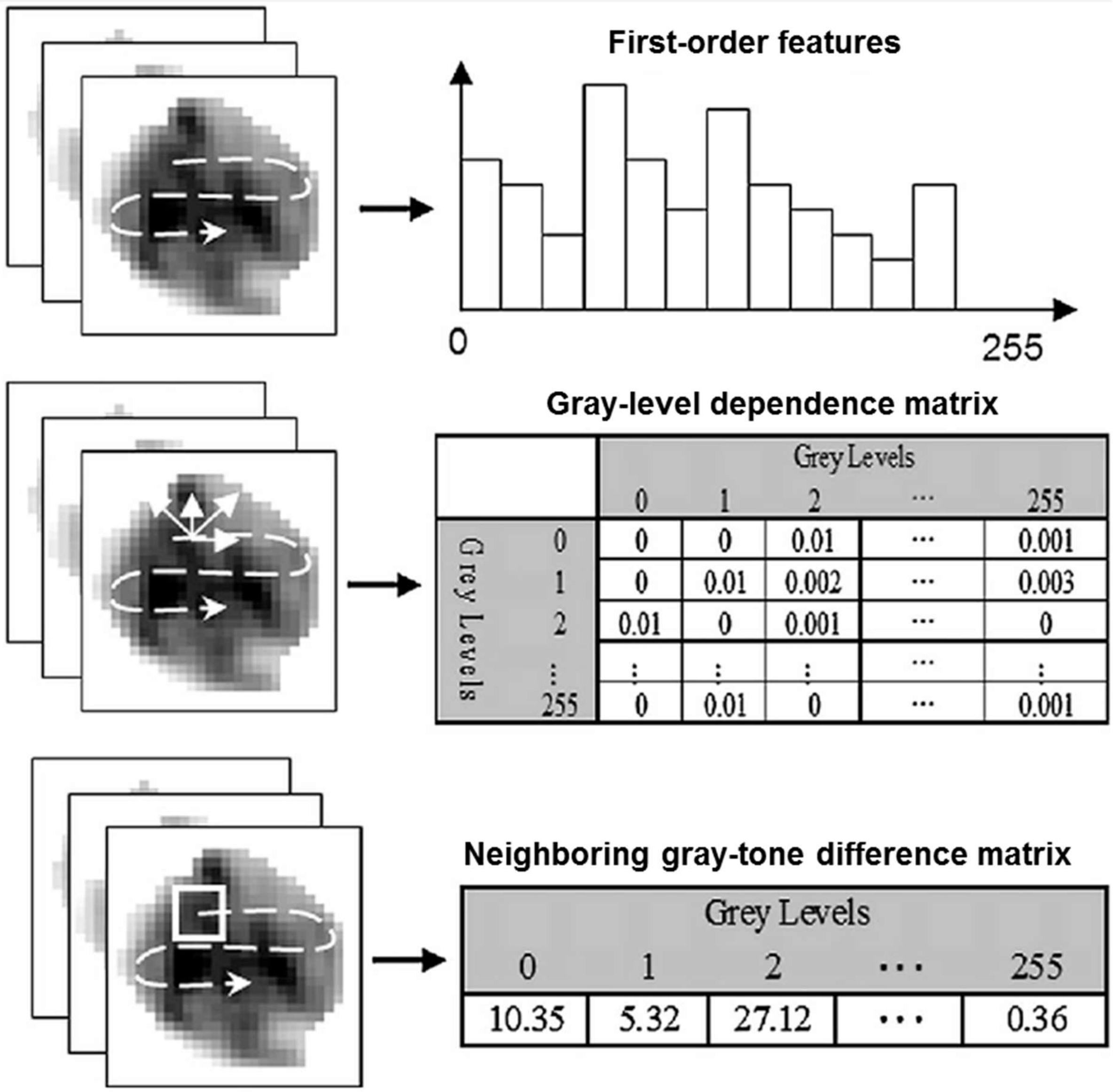


**Figure S1.** Schematic representation of the extraction of first-order features (top), gray-level dependence matrix (GLDM) features (middle), and neighboring gray-tone difference matrix (NGTDM) features (bottom) [1].

**Descriptions of the ML algorithms.**

**Naive Bayes.** Bayes decision theory represents a fundamental statistical approach in pattern classification. This classifier is based on the assumption that the decision problem is formulated in probabilistic terms, and that all relevant probability values are given. Simplified formulated, the probability that a pattern belongs to a given class is determined.

**Logistic regression**. Logistic regression represents a statistical model employing the logistic function for modeling a binary dependent variable. It unveils the relationship between the dependent variable and one or more independent variables based on estimating probabilities employing the logistic regression equation.

**Support vector machine (SVM).** SVMs are supervised learning models for classification purposes and regression analysis. Applied to a two-class problem. they map the training data such as to maximize the width of the gap between the two classes. They can be performed both linear and nonlinear classification. Non-linear classification is achieved based on the kernel trick, implicitly mapping their inputs into high-dimensional feature spaces Kernels can be polynomial, sigmoid or gaussian.

**k-nearest neighbor (kNN).** kNN is a supervised classifier. This algorithm stores all available patterns and classifies new patterns based on a similarity measure (e.g., distance functions) and majority class of the patterns in the specified number of neighbors.

**Decision tree (DT).** Decision tree represent a nonlinear multistage classifier in which classes are rejected over a sequence of decisions until a finally accepted class is reached. This means that the feature space is split sequentially in specific regions which correspond to the classes. Each feature vector traverses an existing tree based on a sequence of decisions and follows a path of nodes until it reaches the region where it belongs. In other words, the correct class corresponding to a feature vector is determined by searching a tree–based decision system. This classification scheme is extremely beneficial when a large number of classes is given. The most popular decision trees are binary decision trees.

**Multilayer perceptron (MLP).** The architecture of the MLP is completely defined by an input layer, one or more hidden layers, and an output layer. The input data is processed by the MLP in a forward direction, passing through each single layer. The training of the network is accomplished based on a supervised learning technique (backpropagation) that requires given input-output data pairs.

**Adaptive boosting (AdaBoost).** AdaBoost is an ensemble learning method and can be used in conjunction with many other types of learning algorithms to improve performance. The output of the other learning algorithms ('weak learners') is combined into a weighted sum that represents the final output of the boosted classifier. AdaBoost is adaptive in the sense that subsequent weak learners are tweaked in favor of those instances misclassified by previous classifiers. AdaBoost typically combines weak learners (such as decision stumps) but can also effectively combine strong learners (such as deep decision trees).

**Random forest.** The random forest is an ensemble method composed of many smaller models. The classification and prediction is achieved by combining the outputs of these smaller models which are usually classification and regression trees (CART). CART operates based on a repeated partitioning of the input data in order to estimate the conditional distribution of a response (output class) for a given set of feature variables. The algorithm implements a binary

decision tree where every single feature of the input is considered as a candidate for the split. Binary decision trees are nonlinear multistage classifiers. This classification system operates by searching a tree-based decision system. The trees are combined to a forest based on bagging. To avoid overfitting, each model is fitted only to a sample of the same size as the original input data but selected with replacement. This sample technique is known as the bootstrap sample.

**Bootstrap aggregating (bagging).** Bagging is an ensemble machine learning algorithm. It is designed to improve stability and accuracy of machine learning algorithms for both classification and regression. Bagging is commonly used to reduce variance within a noisy dataset and helps to avoid overfitting. Bagging is composed of two parts: bootstrapping and aggregation. Bootstrapping is a sampling method, where a sample is randomly chosen out of a set, using the replacement method. The learning algorithm is then run on the samples selected. The outputs from all the separate models are aggregated into a single prediction as part of the final model. Bagging is usually applied to decision tree methods but can also be used with any type of machine learning method.

## Reference

1 Xu, R., Kido, S., Suga, K., Hirano, Y., Tachibana, R., Muramatsu, K.,Chagawa, K., Tanaka, S.Texture analysis on 18F-FDG PET/CT images to differentiate malignant and benign bone and soft-tissue lesions. *Ann. Nucl. Med.* **2014**, *28*, 926–935, https://doi.org/10.1007/s12149-014-0895-9
